# New Insights into the Molecular Structure of Tear Film Lipids Revealed by Surface X-ray Scattering

**DOI:** 10.1021/acs.jpclett.3c02958

**Published:** 2024-01-03

**Authors:** Ryan M. Trevorah, Mira Viljanen, Tuomo Viitaja, Henrik Stubb, Julia Sevón, Oleg Konovalov, Maciej Jankowski, Philippe Fontaine, Arnaud Hemmerle, Jan-Erik Raitanen, Filip S. Ekholm, Kirsi J. Svedström

**Affiliations:** † Department of Physics, University of Helsinki, P.O. Box 64, FI-00014 Helsinki, Finland; ‡ Department of Chemistry, University of Helsinki, P.O. Box 55, FI-00014 Helsinki, Finland; § Ophthalmology, University of Helsinki and Helsinki University Hospital, Haartmaninkatu 8, FI-00290 Helsinki, Finland; ∥ The European Synchrotron Radiation Facility - ESRF, 71 Avenue des Martyrs, CS 40220, Grenoble Cedex 9 38043, France; ⊥ Synchrotron SOLEIL, L’Orme des Merisiers, Départementale 128, 91190 Saint-Aubin, France

## Abstract

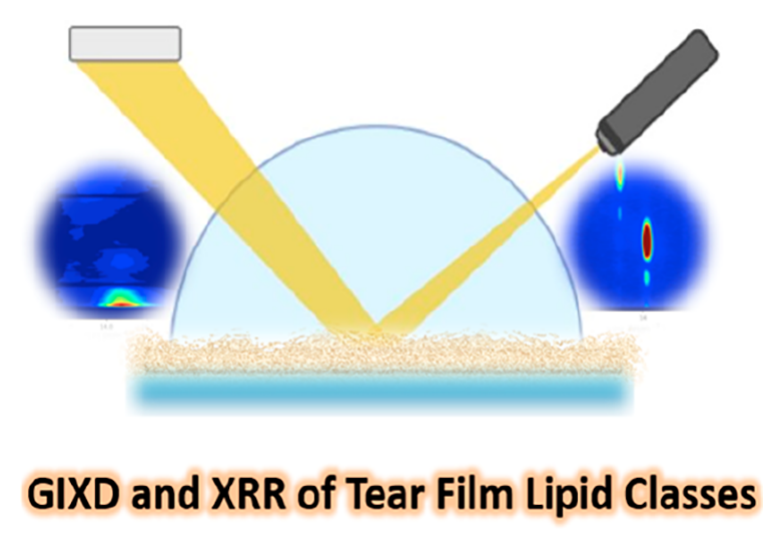

The tear film lipid layer (TFLL) is a unique biological membrane that serves a pivotal role in the maintenance of ocular surface health. Reaching an overarching understanding of the functional principle of the TFLL has been hampered by a lack of insights into the structural and functional roles played by individual lipid classes. To bridge this knowledge gap, we herein focus on studying films formed by principal lipid classes by surface scattering methods. Through grazing incidence X-ray diffraction and X-ray reflectivity studies, we reveal quantitative data about the lattice distances, molecular tilt angles, and mono/multilayer thickness and density profiles for central TFLL lipid classes under close to simulated physiological conditions. In addition, we discuss the correlation of the results to those obtained previously with the natural lipid composition of meibum.

The tear film lipid layer (TFLL) is a complex lipid composition secreted by meibomian glands. The ≥200 lipids that form the core of this composition make up the outermost layer of the tear film, where they serve important roles such as maintaining an optimal surface tension thus preventing tears from spilling over, generating a smooth refractive surface required for clear vision, and moderating the rate of evaporation of aqueous tear fluid.^1−3^ These properties arise through the collaborative action of the individual lipid classes and species, and a correlation between dysfunctions and the development of ocular surface diseases has been noted.^4,5^ Nevertheless, the molecular architecture and functioning principle of an intact TFLL have remained subjects of debate within the community for decades.^6−9^ Studies of the complex natural TFLL have been unable to identify the contributions of individual lipid species, while studies of individual lipid classes have been hampered by limited access to the unique lipid species found in human meibum. To provide the foundation for improving our molecular level understanding of the organization and function of the TFLL, we consider that complementing the previous “top-down” approaches through which the structural and functional properties of meibum have been assessed with a “bottom-up” approach supplying detailed insights into the biophysical and structural profiles of individual lipid components is a must.

In our research program, we have been focusing on the chemical synthesis and biophysical profiling of individual TFLL lipid classes and species.^10−12^ Herein, we open up a new venture by exploring in depth the structural features of films formed by representative TFLL lipids through the use of advanced synchrotron surface scattering techniques: grazing incidence X-ray diffraction (GIXD) and X-ray reflectivity (XRR). Our aim was to assess whether we could provide important quantitative data about the crystal lattice, tilt angles, mono/multilayer thickness, and density profiles of lipid films at the air–water surface to set the foundation for the future assessment of molecular libraries and TFLL-mimicking compositions. In this pioneering work, one representative TFLL lipid from each of the wax ester (WE), cholesteryl ester (CE), *O*-acyl-w-hydroxy fatty acid (OAHFA), and diester (DiE) categories was chosen. These were (21*Z*)-29-oleoyloxynonacos-21-enoic acid (29:1/18:1-OAHFA), (21*Z*)-1,29-dioleoyloxynonacos-21-ene (18:1/29:1/18:1-DiE), cholesteryl 24-methylpentacosanoate (*iso*-CE), and 24-methylpentacosyl oleate (*iso*-WE) (Figure 1). In addition to covering both nonpolar (WEs and CEs) and polar (in the context of TFLL lipids^13^) lipids (OAHFAs and DiEs), these species represent the major lipid classes found in the TFLL as it contains approximately 40–45% WEs, 40–45% CEs, 3–5% OAHFAs, and 6–10% DiEs.

**Figure 1:**
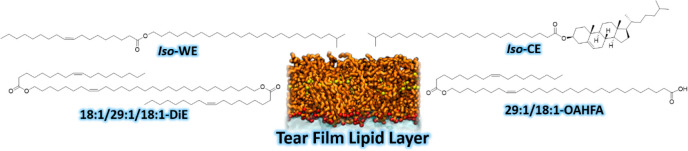
Chemical structures of the four representative TFLL lipid species studied.

In more detail, the endogenous human tear film OAHFAs make up a group of lipids featuring parent carbon-chain lengths in the range of C_18:1_–C_34:1_ with oleic acid (C_18:1_) serving as the most common acyl group.^14^ The 29:1/18:1-OAHFA used as our representative OAHFA corresponds to the weighted average of TFLL OAHFAs reported in a recent lipidomic study.^15^ The type II DiEs are thought to follow trends similar to those of the OAHFAs, and therefore, 18:1/29:1/18:1-DiE is a good representative of this category. The endogenous human WEs and CEs are more complex as they exist in straight-chain, *iso*-branched and *anteiso*-branched forms.^16^ The rich structural diversity displayed cannot be accurately recaptured by a single representative lipid species, and thus, we opted to use the most abundant species. The *iso*-CE chosen accounts for roughly 10–14% of the total TFLL CEs, and the *iso*-WE for 13–20% of the total TFLL WEs. We recently reported on the chemical synthesis and biophysical profiles of these species.^12^ In addition, the wide-angle X-ray scattering data revealed the crystalline structure of the samples in their bulk state.^12^ However, a synchrotron light source is needed to enable surface scattering methods that can reveal the detailed structural properties of these lipid species at the air–aqueous interface.

In the natural environment, the TFLL forms on top of the aqueous tear film layer. To mimic the conditions at the ocular surface, synchrotron GIXD and XRR studies were performed on a Langmuir trough setup specifically tailored for the purpose. The aqueous phase matched the pH and electrolyte concentration of the aqueous tear film layer (pH 7.4, electrolyte concentrations of 137 mM NaCl, 10 mM phosphate, and 2.7 mM KCl), and the in-plane two-dimensional (2D) structure and lamellar layer thickness of the films formed by the representative TFLL lipids were analyzed in the temperature range of 25–35 °C and pressure range of 10–40 mN/m. When the instrumentation allowed, physiological ocular surface temperatures and pressures were employed (∼35 °C and ∼25–35 mN/m, respectively); however, the maximum temperature permitted at one of the synchrotron beamlines was 30 °C, and the low collapse pressure of *iso*-WE and 18:1/29:1/18:1-DiE films required studies at lower surface pressures and temperatures to enable assessment of film structure by the chosen techniques. Isotherms for *iso*-CE and *iso*-WE are included in Supporting Information Figure 1, and those for 29:1/18:1-OAHFA and 18:1/29:1/18:1-DiE can be found in ref (17).

GIXD is a surface scattering technique uniquely suited to studying the molecular lattice distances and tilt angles of biological films at an interface and thus is central to establishing a link between the in-plane molecular architecture and functional properties. Using synchrotron GIXD, studies of Langmuir monolayers of lipid molecules are enabled.^18,19^ From a TFLL perspective, this technique is most valuable in providing insights into the structural basis of the polar lipid layer that resides at the aqueous interface. With access to four distinct TFLL lipid classes, we decided to probe all four individual species by this method. The experimental GIXD data were fitted with Gaussian functions, which enabled the calculation of average coherence lengths, tilt angles, and lattice parameters. A summary of the results is provided in Table 1. The previous GIXD study performed by Leiske et al. on human meibum^8^ gave us a sound reference point for the more complex natural composition, although they did not report the composition of their meibum samples so it is unclear what the relative proportions of the different lipid classes actually were. Our earlier biophysical profiling work^12^ on the individual TFLL lipid species provided important insights into the surface behavior of these lipid species over the range of 10–40 mN/m at 35 °C.

**Table 1:** Summary of the GIXD Results, Including Lattice Parameters (*a* and *b*), Molecular Tilt Angles, and Average In-Plane Coherence Lengths (*B*)^a^

TFLL lipid [lattice type, temperature (°C), pressure (mN/m)]	*a* (Å)	*b* (Å)	tilt angle (deg)	*B* (in plane) (Å)
29:1/18:1-OAHFA (*NN*, 35, 30)	5.3 ± 0.01	8.9 ± 0.05	32 ± 0.5	460 ± 10
18:1/29:1/18:1-DiE (*NN*, 25, 30)	5.3 ± 0.2	9.3 ± 0.4	44 ± 1.5	320 ± 40
*iso*-CE (not possible, 3D crystals)	–	–	–	–
*iso*-WE (*NNN*, 30, 10)	5.0 ± 0.01	9.6 ± 0.05	39 ± 0.5	200 ± 10
meibum (*NNN*, 35, 18), weak (secondary) phase^8^	5.03 ± 0.04	9.99 ± 0.14	23.39 ± 0.14	101.7 ± 15.3
meibum (*NNN*, 35, 18), strong (main) phase^8^	5.02 ± 0.01	9.30 ± 0.11	24.61 ± 0.37	161.9 ± 14.7

a The error margins given for DiE are larger because the reflections were only partially detected. For comparison, the literature values for human meibum determined by Leiske et al.^8^ are shown (full measurement series included in the Supporting Information). *NN* = nearest-neighbor tilted phase. *NNN* = next-nearest-neighbor tilted phase.

As the OAHFAs have been identified as one of the principal lipid classes that contribute to the polar lipid layer, we began by assessing the properties of 29:1/18:1-OAHFA. In line with our earlier observations, the 29:1/18:1-OAHFA species did not give rise to interesting molecular structures at low surface pressures (<10 mN/m) as it exists in a liquid phase. Increasing the surface pressure led to the gradual transition to a solid phase. Under physiological conditions (35 °C and 30 mN/m), we observed a strong on-axis peak at *q*_*xy*_ = 14.1 nm^–1^ and another weaker peak at *q*_*xy*_ = 13.9 nm^–1^ and *q*_*z*_ = 7.6 nm^–1^ (Figure 2A). On the basis of this GIXD pattern, the OAHFAs adopt a structure of the *NN* lattice type. On a general level, some similarities and differences were observed compared to meibum. At surface pressures of <7 mN/m and 35 °C, meibum exists in a liquid phase and at physiological surface pressures in a solid phase^8^ in line with our observations on 29:1/18:1-OAHFA. While the *NN* lattice type of 29:1/18:1-OAHFA was distinct from the *NNN* lattice type reported for meibum,^8^ it was in good agreement with earlier reports on fatty acids.^18^ We also noted that the lattice parameters (lattice distances *a* and *b*) for meibum and 29:1/18:1-OAHFA were in good agreement. The exceptions were the tilt angles that deviated by ∼10° and the in-plane coherence length that was twice as long for the individual OAHFA as for meibum.^8^ Nevertheless, the in-plane coherence length, which corresponds to the average width/size of the crystalline areas of the film, fluctuated between the distinct measurements, and a consistent trend in relation to surface temperature and pressure could not be determined. This would mean that the sizes of the crystalline area are neither constant nor dependent on the pressure and/or temperature. However, all of the coherence lengths were of the same magnitude, so the external accuracy (all of the instrumental effects) might also limit the observation of possible differences. Lattice values *a* and *b* were consistent over the studied surface pressure and temperature range, whereas the tilt angles were found to decrease slightly as a function of an increased surface pressure (see Figure 2D). While deviations between meibum and 29:1/18:1-OAHFA were noted, a perfect match was not expected, as the OAHFAs account for 3–5% of the meibum composition. Moreover, we were intrigued by these proof-of-concept findings, which suggested that the profiles of individual lipid classes may not be directly assessed through studies of complex meibum samples.

**Figure 2:**
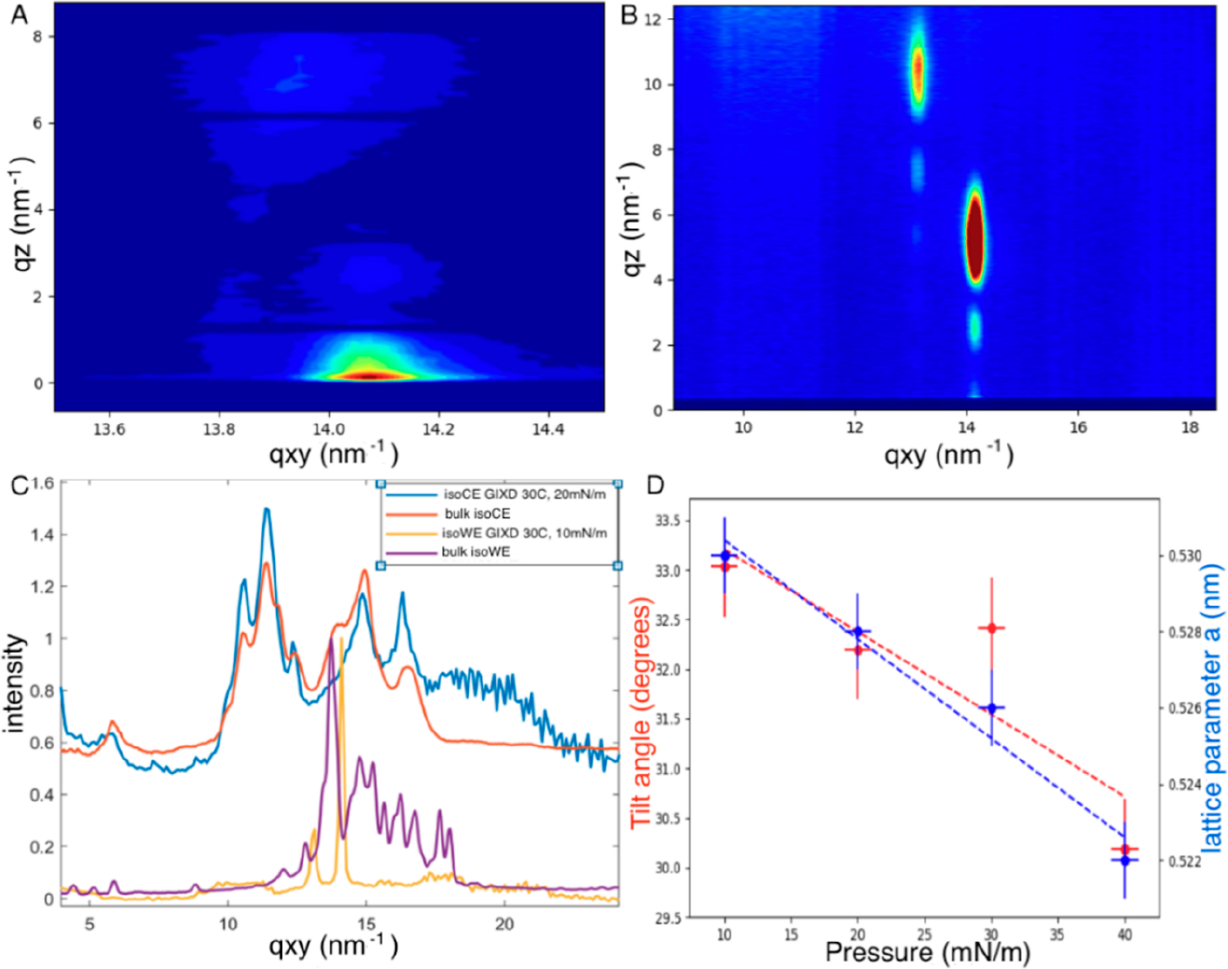
Excerpt of the biophysical results. (A) 2D GIXD of 29:1/18:1-OAHFA illustrating a typical *NN* lattice type (*P* = 20 mN/m). (B) 2D GIXD pattern of *iso*-WE displaying the *NNN* lattice type (*P* = 10 mN/m). (C) Comparison of GIXD spectra for both *iso*-CE and *iso*-WE in bulk form and as measured in the Langmuir trough environment. (D) Illustration of changes in the molecular tilt angle and lattice parameter *a* as a function of increasing surface pressure for the 29-OAHFA sample. Note that dotted lines are simple linear fits, intended as a guide for the eye.

In addition, we assessed the structural properties of the second polar lipid in our series, 18:1/29:1/18:1-DiE. On the basis of our recent report, films of this diester species exist in the liquid phase and collapse at a surface pressure of 1.5 mN/m at 35 °C.^10^ Thus, studies under physiological conditions were not possible. To assess whether this species could be studied using GIXD, we decided to decrease the temperature to 25 °C while keeping the surface pressure at 30 mN/m. Under these conditions, the lattice type and parameters of the 18:1/29:1/18:1-DiE species could be uncovered, however, with relatively large error margins, because the other diffraction peak observed (referring to the *NN* lattice) was only partially covered by the detector area (Supporting Information Figure 3). While the type II diester was not assessed under physiological conditions, the *NN* lattice type was in line with that reported for 29:1/18:1-OAHFA, the other polar lipid covered in this study. In contrast to those of 29:1/18:1-OAHFA, the tilt angles for the DiE species showed a surface pressure-dependent increase. This opposing behavior of the two polar lipid species is interesting, although the underlying reasons remain unclear. Lattice parameters *a* and *b* were relatively consistent across the range of 10–30 mN/m and similar to those of 29:1/18:1-OAHFA and meibum.^8^ The tilt angles were 12° larger than those for the OAHFAs and 20° larger than those reported for meibum. In contrast to the OAHFA sample, the DiE sample seemed to display a decreasing correlation between an increased surface pressure and in-plane coherence lengths. Due to the limited number of measurement points, additional studies would have been required to determine whether this is a general trend. Nevertheless, we were able to uncover important new insights into the structural properties of solid type II diester films. In addition, we consider mapping the boundaries and limitations of the synchrotron techniques to be an equally important finding as this provides insights into the selection criteria that will allow further refinement of the substrate scope used in future studies.

We next shifted our focus to the nonpolar lipid classes by addressing the structural and surface properties displayed by *iso*-WE and *iso*-CE. In our recent work, we reported bulk WAXS data for *iso*-CE. In addition, our Brewster angle microscopy (BAM) measurements showed that *iso*-CE forms aggregates at physiological surface temperature and pressure.^12^ While these properties were not considered ideal for GIXD studies, we considered that the bulk WAXS data could, in fact, provide valuable insights into the structural contributions of similar species because these are not a result of interactions taking place at the interface. As shown in Figure 2C, our assumptions were proven correct and *iso*-CE retained the same bulk structure also at the air–water interface. The Debye rings observed in the GIXD patterns indicated that *iso*-CE forms three-dimensional (3D) crystallites. These features appear at all pressures studied but become more prominent at higher pressures (Supporting Information Figure 4). We note that of the four TFLL lipid classes studied herein, *iso*-CE was the only one to show this kind of tendency and/or property. A careful review of the literature revealed that the formation of 3D crystallites on the air–aqueous surface has been observed previously for glycolipids^20^ but not in GIXD studies of long-chain CEs.^21^ Considering that the TFLL consists of approximately 40–45% CEs, our findings indicate that *iso*-CEs are likely to contribute to the crystalline phases observed in meibum *in vivo*.

In our recent biophysical profiling of *iso*-WE, we discovered that its behavior is different from that of its straight-chain counterpart. It formed a solid monolayer film with two distinct regions of different thicknesses at surface pressures of <20 mN/m, and the formed film displayed evaporation resistant features.^12^ The straight-chain counterpart, on the contrary, was found to form aggregates. Nevertheless, it must be noted that the Brewster angle microscope employed in the imaging of the films does not enable characterization at the molecular level in a fashion similar to that of GIXD. In contrast to the findings for *iso*-CE, the GIXD studies of *iso*-WE revealed that there is a significant difference between the bulk state structure and the one that forms when *iso*-WE spreads on an aqueous surface (Figure 2C). Thus, corroborating our earlier indications, *iso*-WE forms a film with a characteristic 2D crystalline structure. Studies at ocular surface temperatures and pressures were not possible due to the limitations set by the low collapse pressure of *iso*-WE (observed at ∼14.5 mN/m). The lattice type of *iso*-WE was determined to be *NNN*, and lattice parameters *a* and *b* were consistent over the studied temperature and surface pressure ranges. Lattice parameters *a* and *b* were in the same range as those of the other TFLL lipid species studied (Table 1) and the literature values of meibum.^8^ The *NNN* lattice type corresponds to that reported previously for meibum. In a fashion similar to that for the type II diester, the tilt angles were found to increase as a function of increased surface pressure. The tilt angles were similar to those of the type II diester and slightly larger than the corresponding values of the OAHFA. The in-plane coherence length fluctuated between the measurements, and sound trends could not be observed, like in similar studies of 29:1/18:1-OAHFA.

Altogether, we note that across the studied surface pressure and surface temperature ranges the lattice parameters (*a* and *b*) were relatively consistent for all studied TFLL lipid classes and were in line with the previous report on meibum. This is an important finding considering that meibum is comprised of many individual lipid species, each of which contributes to the overall physical properties of bulk meibum. The lattice type of the 29:1/18:1-OAHFA and 18:1/29:1/18:1-DiE was found to differ from that of the other TFLL lipid species and that reported for meibum. The tilt angles for all lipid classes were likewise considerably larger than those reported for meibum, and the opposing trends in the surface pressure dependence of the TFLL lipid classes studied were observed. There can be two main reasons for these deviations. Either the subtle structural contributions of individual species may be difficult to uncover in studies of natural meibum, or the properties change as a result of interactions between different lipid classes and species. More work with a wider substrate library and carefully composed mixtures will be required to provide an answer to these questions. Here, we continued by assessing the two most promising lipid species (29:1/18:1-OAHFA and *iso*-WE) by synchrotron XRR method.

The GIXD and XRR studies are often performed as a pair, as they provide complementary insights into the structure of films at an interface: GIXD providing information about the in-plane structures and XRR providing information perpendicular to the plane (i.e., film profile).^22^ The main advantage of the advanced XRR synchrotron technique is that it can be used to address important surface properties such as film thickness and density profiles at gas–liquid, liquid–liquid, or solid–liquid interfaces. These properties are relevant from the TFLL perspective as they may help explain if the principal TFLL lipid classes tend to form monolayer or multilayer structures at the aqueous interface. This is an important factor when it comes to understanding the molecular architecture of TFLL and its possible distinct domains. In this work, we chose to focus on 29:1/18:1-OAHFA and *iso*-WE. In addition to having one representative of the polar and nonpolar lipid classes, these two species seemed to be the most suitable candidates of our set on the basis of the GIXD results. In addition, *iso*-CE was characterized by XRR to confirm the interesting observations seen by GIXD.

The XRR studies of 29:1/18:1-OAHFA were performed at 30 °C (maximum allowed temperature at the Langmuir setup used) over the surface pressure range of 5–30 mN/m (Figure 3). At low surface pressures (<10 mN/m), no significant structure was observed in accordance with the GIXD findings, our recent literature reports,^12^ and the previous reports on meibum.^8^ At 10 mN/m, some structure was observed, but the monolayer was not dense enough to be reliably modeled. Nevertheless, performing the XRR studies at selected surface pressures enabled the identification of the correlation between the structured film formation and surface pressure. The formation of a structured monolayer above 10 mN/m was eminent on the basis of the clearer oscillations in the XRR spectra. Overall, the results were in line with the GIXD data and our earlier assessments, in which we imaged the film by BAM. However, here we were able to go beyond the assessments performed earlier. First, the solid monolayer structure could be modeled using a two-slab model and the Refl1D software package.^23^ The scattering length densities and layer thicknesses were modeled for both the head and tail groups of 29:1/18:1-OAHFA, and the best fit to our data translated into a total monolayer thickness of 54.1 ± 1.0 Å for the spectrum collected at 30 mN/m and 30 °C (see the Supporting Information for more details). Thus, we can conclude that the average TFLL OAHFA can form a solid monolayer at the aqueous interface with a layer thickness of 54.1 ± 1.0 Å.

**Figure 3:**
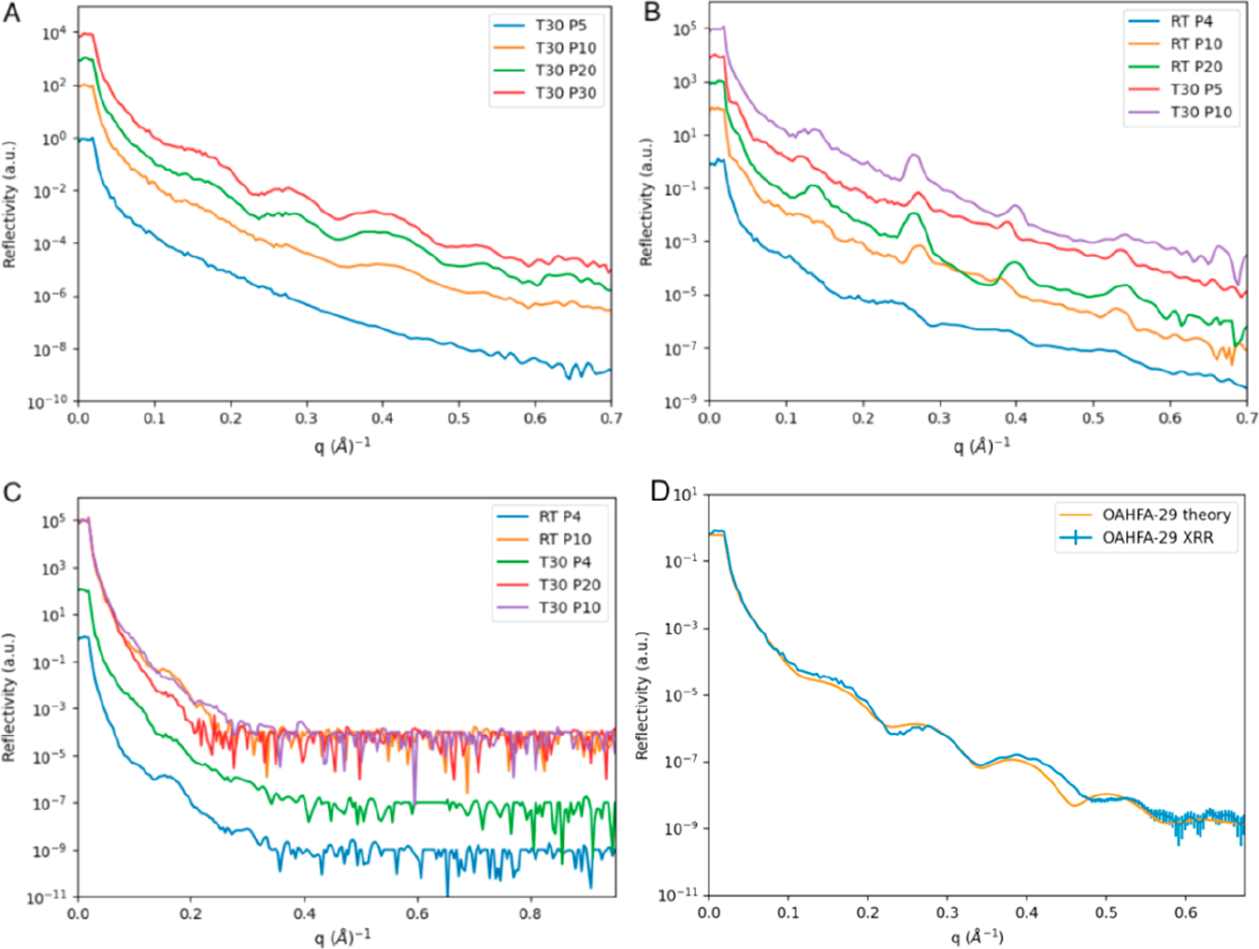
Excerpt of the XRR studies. (A) Comparison of XRR spectra for 29:1/18:1-OAHFA as a function of pressure. (B) Temperature and pressure series XRR spectra for *iso*-WE. (C) Temperature and pressure series XRR spectra for *iso*-CE. (D) Illustration of XRR spectra with the best-fit theory model overplotted for 29:1/18:1-OAHFA. Note that some spectra have been scaled in the *y* dimension for the sake of clarity. Legend: RT = room temperature (∼25 °C), T30 = 30 °C, P4 = surface pressure of 4 mN/m.

We continued by studying the film behavior of *iso*-WE. To avoid the collapse of the *iso*-WE film, we performed the studies at surface pressures of 5–10 mN/m at 30 °C (as well as complementary studies at 4–20 mN/m at 25 °C). The behavior of the *iso*-WE film was different from that of 29:1/18:1-OAHFA (see panels A and B of Figure 3). The *iso*-WE XRR spectra did not display an oscillatory trend like that for 29:1/18:1-OAHFA; instead, periodic Bragg peaks were observed, which indicates the formation of a lamellar multilayer^8,22^ (Supporting Information Figure 2). These results allowed us to revise our earlier findings on the biophysical profile of *iso*-WE. In more detail, we noted in BAM images that layers with distinct intensities formed but we were not able to deduce if these were multilayers or monolayers with accompanying aggregates. Thus, the XRR data afforded welcome yields and indisputable insights into the behavior of *iso*-WE. From the *d* spacing determined by the Bragg peaks, a value of 46.9 ± 1.0 Å was calculated as the lamellar spacing. This is an interesting observation when compared to the results reported by Leiske et al. In more detail, they observed the formation of multilayers above 15 mN/m with *d* spacing values of 50 Å for human meibum using XRR.^8^ This is very close to the values uncovered for both the total thickness of the 29:1/18:1-OAHFA monolayer and the lamellar distance observed in a multilayer formed by *iso*-WE. In addition to 29:1/18:1-OAHFA and *iso*-WE, *iso*-CE was measured by XRR just to confirm its GIXD results. The very fast decay of the reflectivity intensity observed in the XRR curves (Figure 3C) corresponds to a very high interface roughness. This is in line with the observation of the formation of 3D crystallites by GIXD.

While additional studies will be required to provide a more comprehensive perspective on the topic, it is reassuring to note that both our study on the individual components and the studies on meibum itself indicate that structuring of multilayers observed in the complex natural composition follows a pattern that can be recognized on the basis of the individual components assessed herein. Moreover, when the lamellar distances (long spacing values) for the main phase and secondary phase for meibum in its bulk state are considered (49 and 110 Å, respectively),^24^ the first of these values resembles the distance identified for 29:1/18:1-OAHFA and *iso*-WE at the aqueous interface and the latter resembles the long spacings identified for *iso*-WE and *iso*-CE in their bulk states (85 and 105 Å, respectively).^12^

Thus, it seems clear that our pioneering work devoted to understanding the structural contributions of individual TFLL lipid classes to the molecular architecture of meibum shows significant potential. To the best of our knowledge, this study is the first in which the potential and limitations of the advanced synchrotron techniques GIXD and XRR are mapped, with the goal of establishing an assessment platform for uncovering the structural features of the characteristic TFLL lipid classes. While we were able to gain important new insights into all four TFLL lipid classes included in the study, the OAHFA and WE species seemed to be the most well-suited group for the experimental setup employed. Several properties (especially the lattice parameters and layer and lamellar thicknesses) were found to be in good agreement with those reported earlier for meibum, but differences were likewise noted.

Through the GIXD studies, we were able to identify a distinct lattice type for the polar OAHFA and DiE species compared with that of the nonpolar lipid classes studied. Moreover, the tilt angles were found to be considerably larger for all of the studied lipid classes than those reported for meibum, and an opposing relationship between enlargement and/or retraction of tilt angles as a function of surface pressure was noted for the OAHFA and WE/DiE studied. While the differences uncovered may be a result of the more detailed assessment enabled by studying individual TFLL lipids, they may likewise be related to structural reorganizations occurring in mixed compositions. Through the XRR studies, we were able to prove that the nonpolar WEs form multilayers and the polar OAHFAs monolayers. Moreover, the lamellar spacing distances uncovered for the individual TFLL lipid classes were found to match earlier observations reported in human meibum. Intrigued by the successful proof-of-concept study reported herein, we will continue on our “bottom-up” approach to understanding the molecular architecture of meibum by assessing the profiles of compound libraries and carefully selected TFLL-mimicking compositions. These studies will be required to further address the structural contributions of distinct TFLL lipid classes and how structural deviations within and interactions between lipid classes affect their organizational roles within the TFLL context.

## Supporting Information Available

Information about materials, experimental details, and supporting data (PDF)
