# Supplementary material for: New Insights into the Molecular Structure of Tear Film Lipids Revealed by Surface X-ray Scattering
Source: J Phys Chem Lett. 2024 Jan 3;15(1):316–22. doi: 10.1021/acs.jpclett.3c02958 (PMC10788950; doi:10.1021/acs.jpclett.3c02958)
Supplement: Supplementary file 1 — jz3c02958_si_001.pdf [file jz3c02958_si_001.pdf]

Supporting Information

New Insights into the Molecular Structure of Tear Film Lipids Revealed by Surface X-ray Scattering

Ryan M. Trevorah,[1] Mira Viljanen,[1] Tuomo Viitaja,[2,3] Henrik Stubb,[2] Julia Sevón,[2] Oleg Konovalov,[4] Maciej Jankowski,[4] Philippe Fontaine,[5] Arnaud Hemmerle,[5] Jan-Erik Raitanen,[2] Filip S. Ekholm,[2] and Kirsi J. Svedström*,[1]

[1] Department of Physics, University of Helsinki, P.O. Box 64, FI-00014 Helsinki, Finland

[2] Department of Chemistry, University of Helsinki, P.O. Box 55, FI-00014 Helsinki, Finland

[3] Ophthalmology, University of Helsinki and Helsinki University Hospital, Haartmaninkatu 8, FI-00290 Helsinki, Finland

[4] The European Synchrotron Radiation Facility – ESRF, 71 Avenue des Martyrs, CS 40220, Grenoble Cedex 9 38043, France

[5] Synchrotron SOLEIL, L'Orme des Merisiers, Départementale 128, 91190, Saint-Aubin, France

Corresponding Author

*kirsi.svedstrom@helsinki.fi

Table of contents

1. Materials
2. Grazing Incidence X-ray Diffraction
3. X-ray Reflectivity
4. Supporting Results

1. Materials

Solvents and reagents utilized in the studies were purchased from commercial sources. The TFLL lipids, namely: (21*Z*)-29-oleoyloxynonacos-21-enoic acid (**29:1/18:1-OAHFA**), (21*Z*)-1,29-dioleoyloxynonacos-21-ene (**18:1/29:1/18:1-DiE**), cholesteryl 24-methylpentacosanoate (***iso*-CE**) and 24-methylpentacosyl oleate (***iso*-WE**), were synthesized and characterized as described in our recent work [1].

2. Grazing incidence x-ray diffraction

Instrumentation: GIXD data were collected at the ID10 beamline of the European Synchrotron Radiation Facility (ESRF) in Grenoble, France. The incident monochromatic beam energy was 22.0 keV. The beam incidence angle was 0.045 degrees (approx. 0.79 mrad). A Dectris Mythen 2K detector was used with a resolution of 200 pixels per degree. The beam size was 10 µm in the vertical dimension and 34 µm in the horizontal dimension. Attenuators were employed as sample damage was observed when exposed to the full beam flux. Oxidation was minimized by continuously pumping helium into the Langmuir trough environment during measurements. GIXD data were also collected at the SIRIUS beamline of the SOLEIL Synchrotron in Paris, France. The incident monochromatic beam energy was 8.0 keV. The beam incidence angle was 2.03 mrad. The beam size was 110 µm in the vertical dimension and 2 mm in the horizontal dimension. Attenuators were employed as sample damage was observed when exposed to the full beam flux. Oxidation was minimized by continuously pumping helium into the Langmuir trough environment during measurements. A 2D Pilatus3 1M detector (Dectris, Switzerland) was used associated with a Soller collimator leading to a resolution of about $0.07 nm^{-1}$.

Langmuir trough

ID10 is equipped with a Langmuir trough measuring 170 mm x 438 mm and with a depth of 3 mm. A single moveable barrier controls surface pressure which is measured using a Wilhelmy balance (Surface Pressure Sensor Model PS4, Nima Technology Ltd). When in use, the trough is sealed with a cover and filled with helium. Temperature is control by a thermostated bath and an active vibration isolation system (MOD-2 S, Halcyonics) was used. Standard PBS buffer solution was used as the substrate.

SIRIUS is equipped with a homemade Langmuir trough of $700 cm^2$, and the pressure is measured using a Wilhelmy balance (Riegler and Kirstein GmbH). The mean molecular area is controlled by a single moveable barrier with a maximum compression ratio of 3.8.

Data analysis: Observed features in the GIXD scans were fitted with Gaussian functions and the peak centroids were used to calculate the coherence length, tilt angle and lattice parameters (*a*,*b*) of each of our samples. The lattice type (i.e., *NN or NNN*) was identified by careful inference through consideration of the position of observed features.

The Scherrer equation can be used to calculate the coherence length, that is related to the average size of the crystalline areas in the film:

$$B_{xy} = 0.9\left(\frac{2\pi}{FWHM_{intrinsic}}\right)$$

For a sample with a rectangular lattice, parameters *a* and *b* can be calculated with the following equations:

$$a = \frac{2\pi}{\frac{1}{2}\left(q_{xy,\{(20)\}}\right)} = \frac{1}{\sqrt{\left(\frac{q^2_{(xy,11)}}{4\pi^2} - \frac{1}{b^2}\right)}}$$

where $q_{x,y\ 20}$ is the peak centroid of the non-degenerate peak and $q_{x,y\ 11}$ is the peak centroid of the degenerate peak in the $q_{x,y}$ plane.

$$b = \frac{2\pi}{\frac{1}{2}\left(q_{xy,\{(02)\}}\right)}$$

Tilt angle can be calculated in the *NNN* case with:

$$\tan\theta_{NNN} = \frac{q_{n,z}}{q_{n,xy}}$$

where *n* and *d* refer to the non-degenerate and degenerate peaks, respectively; and in the *NN* case with:

$$\tan\theta_{NN} = \frac{q_{d,z}}{\sqrt{\left(\left(q_{d,xy}\right)^2 - \frac{q_{n,xy}{}^2}{4}\right)}}$$

3. X-ray reflectivity

Instrumentation: XRR measurements were conducted at beamline ID10 at ESRF. The sample environment was identical as described in the GIXD experimental section. A Maxipix detector (2560 x 256 pixels) was used with a distance of 891.7mm between sample and detector leading to a resolution of 1 degree per 283 pixels.

XRR data analysis: The XRR data were analysed using the Refl1d program[2]. The lipid film structure was modelled using a slab model, i.e., the lipid heads and tails were assumed to form layers with different electron densities. The scattering length density (SLD) profiles were modelled using three parameters per each layer implemented in the model: layer thickness, electron density and roughness, which described the roughness of the interface between the layers.

4. Supporting results

Supporting Table 1. variations in fitted model parameters as a function of temperature and pressure. Tilt angles were not able to be calculated for some samples as the Gaussian peak was beyond the measurement region.

**Sample:**	**a (Å)**	**b (Å)**	**tilt angle (deg)**	**B(in plane) (Å)**
iso-WE RT P4	5.00 ± 0.01	9.59 ± 0.05	39.1 ± 0.5	200 ± 10
iso-WE RT P10	5.01 ± 0.01	9.59 ± 0.05	39.2 ± 0.5	240 ± 10
iso-WE RT P20	5.01 ± 0.01	9.57 ± 0.05	40.0 ± 0.5	240 ± 10
iso-WE T30 P5	5.00 ± 0.01	9.55 ± 0.05	38.0 ± 0.5	404 ± 10
iso-WE T30 P10	5.02 ± 0.01	9.59 ± 0.05	38.7 ± 0.5	200 ± 10
18:1/29:1/18:1-DiE T25 P10	5.29 ± 0.2	9.26 ± 0.4	41.2 ± 1.5	490 ± 40
18:1/29:1/18:1-DiE T25 P20	5.29 ± 0.2	9.27 ± 0.4	42.4 ± 1.5	280 ± 40
18:1/29:1/18:1-DiE T25 P30	5.31 ± 0.2	9.29 ± 0.4	44.1 ± 1.5	320 ± 40
29:1/18:1-OAHFA T25 P5	5.19 ± 0.01	9.17 ± 0.05	38.1 ± 0.5	240 ± 10
29:1/18:1-OAHFA T25 P10	5.16 ± 0.01	9.17 ± 0.05	36.2 ± 0.5	240 ± 10
29:1/18:1-OAHFA T25 P20	5.13 ± 0.01	9.15 ± 0.05	35.6 ± 0.5	200 ± 10
29:1/18:1-OAHFA T25 P30	5.06 ± 0.01	9.11 ± 0.05	34.7 ± 0.5	200 ± 10
29:1/18:1-OAHFA T30 P5	4.91 ± 0.01	9.20 ± 0.05	-	283 ± 10
29:1/18:1-OAHFA T30 P10	5.24 ± 0.01	9.19 ± 0.05	36.7 ± 0.5	300 ± 10
29:1/18:1-OAHFA T30 P20	5.18 ± 0.01	9.07 ± 0.05	33.0 ± 0.5	120 ± 10

29:1/18:1-OAHFA T30 P30	5.14 ± 0.01	9.10 ± 0.05	31.0 ± 0.5	240 ± 10
29:1/18:1-OAHFA T30 P35	5.12 ± 0.01	9.10 ± 0.05	-	160 ± 10
29:1/18:1-OAHFA T35 P10	5.30 ± 0.01	8.92 ± 0.05	33.0 ± 0.5	273 ± 10
29:1/18:1-OAHFA T35 P20	5.28 ± 0.01	8.90 ± 0.05	32.2 ± 0.5	462 ± 10
29:1/18:1-OAHFA T35 P30	5.26 ± 0.01	8.90 ± 0.05	32.4 ± 0.5	462 ± 10
29:1/18:1-OAHFA T35 P40	5.22 ± 0.01	8.93 ± 0.05	30.2 ± 0.5	400 ± 10

Supporting Table 2. Further details of XRR fitted models.

**29:1/18:1-OAHFA, T=30C**		
**Refined Parameter**	Surface pressure	
	20 (mN/m)	30 (mN/m)
Upper layer (tail, interface with air)		
Thickness (Å)	49.6 ± 1.0	49.5 ± 1.0
SLD (x10^6 Å^{-2})	10.90 ± 0.3	10.84 ± 0.3
Roughness (Å)	3.25 ± 0.25	3.78 ± 0.25
Lower Layer (head)		
Thickness (Å)	4.0 ± 1.0	4.63 ± 1.0
SLD (x10^6 Å^{-2})	12.66 ± 0.3	12.80 ± 0.3
Roughness (Å)	2.78 ± 0.25	3.50 ± 0.25
Total film thickness (Å)	53.6 ± 2.5	54.1 ± 2.5

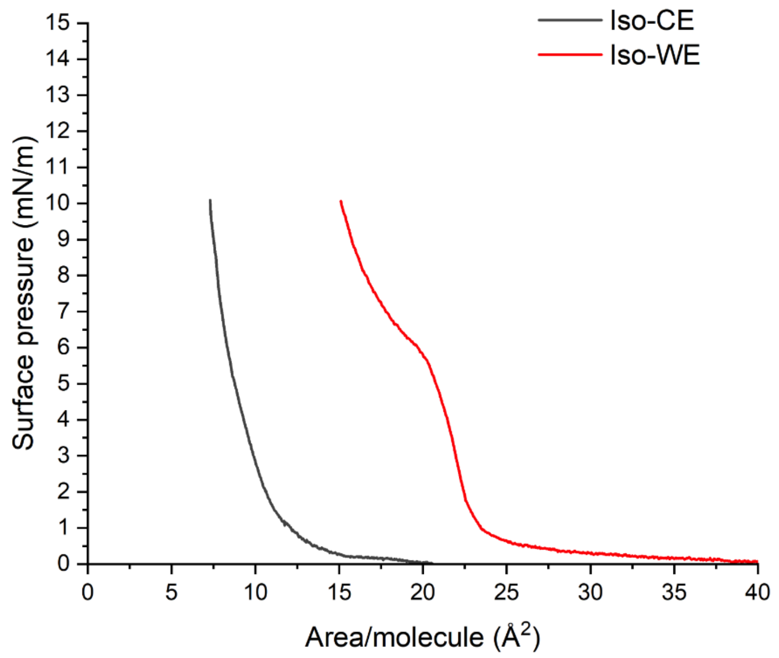

Supporting Information Fig 1: isotherms for *iso*-CE and *iso*-WE. A sharp lift-off is observed at ~22 Å^2/molecule for *iso*-WE and at ~11 Å^2/molecule for *iso*-CE.

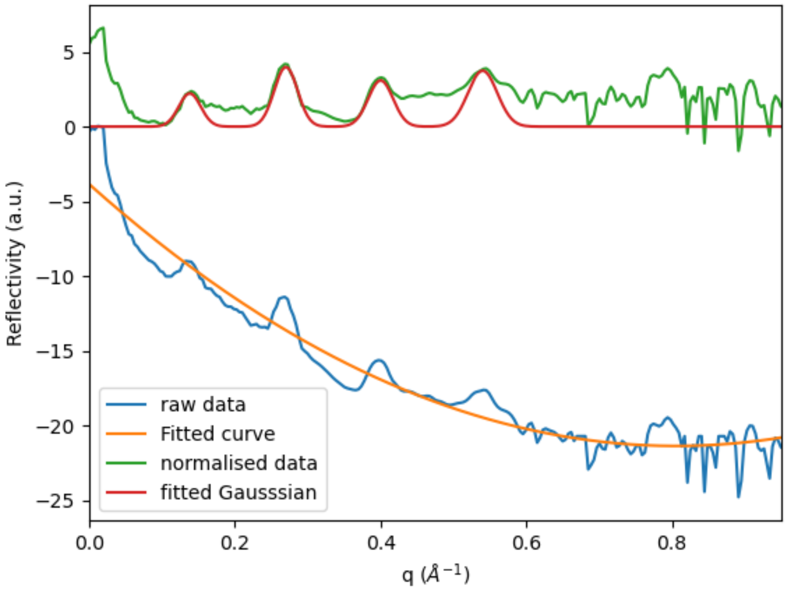

Supporting Information Fig. 2: illustration of data processing to fit the Bragg peaks observed in the *iso*-WE XRR spectra. Peak centroids were fitted at q = [0.14, 0.27, 0.40, 0.54] Å^{-1}. The periodicity was found to be 47 ± 1 Å.

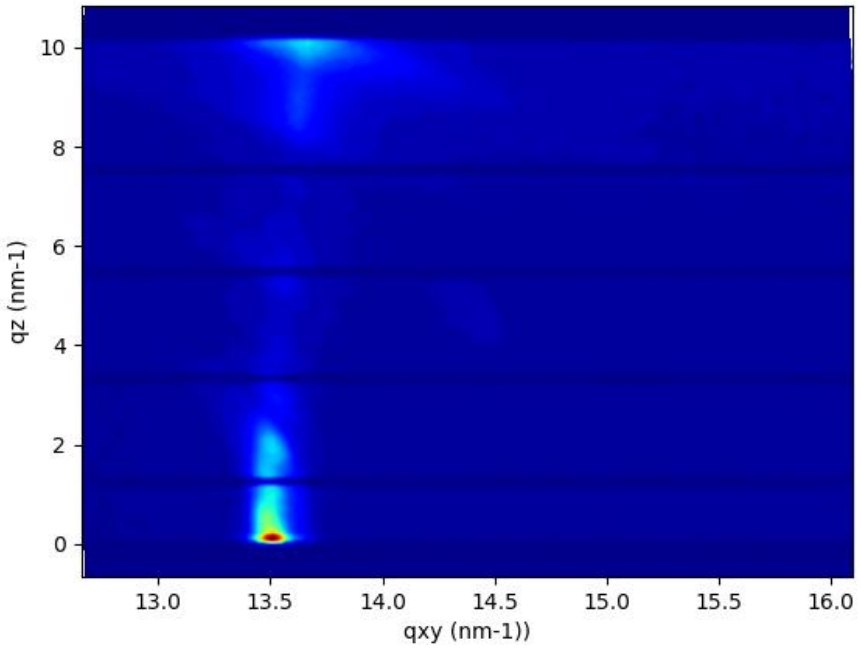

Supporting Information Fig. 3: a 2D GIXD scan of DiE measured at T=25C, P = 30mN/m. An *NN* lattice type is clearly observed, however one peak is beyond the q_z range of the measurement and so could not be fitted to the same accuracy as the other measurements.

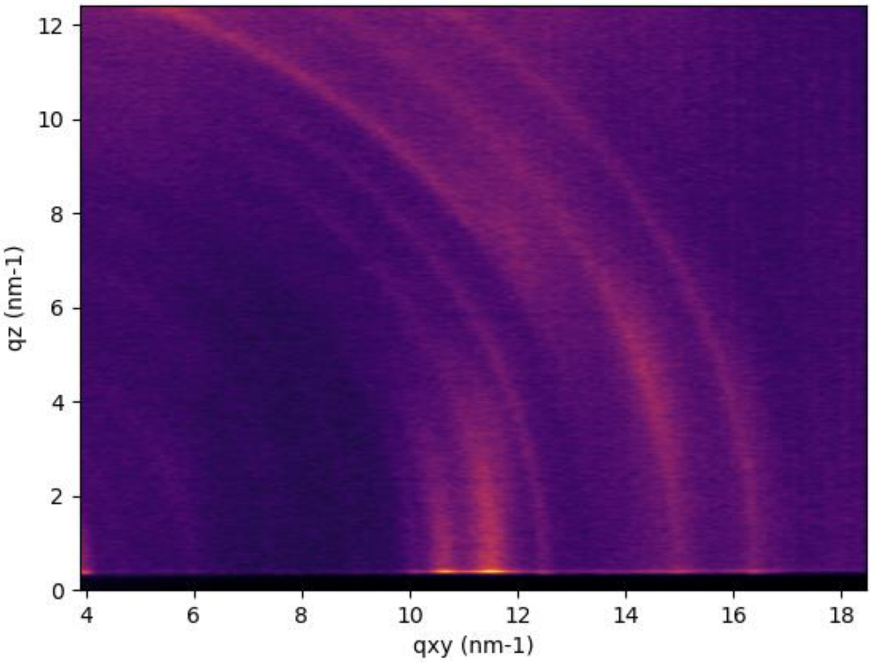

Supporting Information Fig. 4: a 2D GIXD spectrum of *iso*-CE at T = 30C, P = 20mN/m. The circular ring pattern (Debye ring) observed is a clear indication of the formation of 3D crystalline structure.

(1) Viitaja, T.; Raitanen, J. E.; Hynynen, A.; Moilanen, J.; Svedström, K.; Paananen, R. O.; Ekholm, F. S. On the Importance of Chain Branching in Tear Film Lipid Layer Wax and Cholesteryl Esters. *Colloids Surf B Biointerfaces* **2022**, *214*, 112429.
(2) Kienzle, P.A.; Krycka, J.; Patel, N.; Sahin, I. Refl1D (Version 0.8.16) [Computer Software]. College Park, MD: University of Maryland. Retrieved Nov 14, 2023.
